# There is no reliable evidence that providing authors with customized article templates including items from reporting guidelines improves completeness of reporting: the GoodReports randomized trial (GRReaT)

**DOI:** 10.1186/s12874-025-02518-0

**Published:** 2025-03-14

**Authors:** Caroline Struthers, James Harwood, Jennifer Anne de Beyer, Patricia Logullo, Gary S Collins

**Affiliations:** UK EQUATOR Centre, Centre for Statistics in Medicine, NDORMS, University of Oxford, Oxford, UK

**Keywords:** Standards, Reporting guidelines, Education, Reproducibility

## Abstract

**Background:**

Although medical journals endorse reporting guidelines, authors often struggle to find and use the right one for their study type and topic. The UK EQUATOR Centre developed the GoodReports website to direct authors to appropriate guidance. Pilot data suggested that authors did not improve their manuscripts when advised to use a particular reporting guideline by GoodReports.org at journal submission stage. User feedback suggested the checklist format of most reporting guidelines does not encourage use during manuscript writing. We tested whether providing customized reporting guidance within writing templates for use throughout the writing process resulted in clearer and more complete reporting than only giving advice on which reporting guideline to use.

**Design and methods:**

GRReaT was a two-group parallel 1:1 randomized trial with a target sample size of 206. Participants were lead authors at an early stage of writing up a health-related study. Eligible study designs were cohort, cross-sectional, or case-control study, randomized trial, and systematic review. After randomization, the intervention group received an article template including items from the appropriate reporting guideline and links to explanations and examples. The control group received a reporting guideline recommendation and general advice on reporting. Participants sent their completed manuscripts to the GRReaT team before submitting for publication, for completeness of each item in the title, methods, and results section of the corresponding reporting guideline. The primary outcome was reporting completeness against the corresponding reporting guideline. Participants were not blinded to allocation. Assessors were blind to group allocation. As a recruitment incentive, all participants received a feedback report identifying missing or inadequately reported items in these three sections.

**Results:**

Between 9 June 2021 and 30 June 2023, we randomized 130 participants, 65 to the intervention and 65 to the control group. We present findings from the assessment of reporting completeness for the 37 completed manuscripts we received, 18 in the intervention group and 19 in the control group. The mean (standard deviation) proportion of completely reported items from the title, methods, and results sections of the manuscripts (primary outcome) was 0.57 (0.18) in the intervention group and 0.50 (0.17) in the control group. The mean difference between the two groups was 0.069 (95% CI -0.046 to 0.184; *p* = 0.231). In the sensitivity analysis, when partially reported items were counted as completely reported, the mean (standard deviation) proportion of completely reported items was 0.75 (0.15) in the intervention group and 0.71 (0.11) in the control group. The mean difference between the two groups was 0.036 (95% CI -0.127 to 0.055; *p* = 0.423).

**Conclusion:**

As the dropout rate was higher than expected, we did not reach the recruitment target, and the difference between groups was not statistically significant. We therefore found no evidence that providing authors with customized article templates including items from reporting guidelines, increases reporting completeness. We discuss the challenges faced when conducting the trial and suggest how future research testing innovative ways of improving reporting could be designed to improve recruitment and reduce dropouts.

## Background

There is evidence that, although most reputable medical journals endorse the use of reporting guidelines, authors may still find it difficult to identify the most appropriate one and use it effectively to improve the completeness of their article [2]. In 2019, the UK EQUATOR Centre developed an online reporting guideline finder [3]. Responding to prompts, the tool helps researchers identify the most appropriate reporting guideline (e.g., CONSORT [4], STROBE [5], or PRISMA [6]) for their study and delivers an online downloadable checklist. The checklist, hosted on a website, includes links to further information and examples reporting for each item [7]. The checklist intervention was intended to help authors ensure they have included the required information in their manuscript. The checklist can be annotated to indicate where in the manuscript each item has been reported, it can then be submitted to a journal alongside the manuscript. Completed checklists are required by many journals (e.g., BMJ).

However, pilot data collected in collaboration with *BMJ Open* indicated that when a checklist was delivered immediately before submission, it did not lead to authors making significant additions to their manuscripts [1]. The study also collected data on user attitudes and behaviour which indicated that reporting guidance was needed earlier in the writing process. This need was reinforced by a thematic synthesis of barriers to using reporting guidelines [8]. These findings led us to explore writing aid interventions based on reporting guidelines.

A scoping review found a writing aid based on CONSORT (COBWEB) which aimed to improve adherence to CONSORT when writing up a randomized trial report [9]. A 2015 controlled trial of COBWEB invited students to participate in a writing session and complete six sections of a manuscript based on a published protocol [10]. This trial showed COBWEB was effective in improving reporting in controlled conditions. However, a pragmatic trial involving 46 journals investigating the use of a web-based CONSORT checklist intervention (WebCONSORT) delivered after submission to a journal failed to show a beneficial effect [2].

We therefore sought to evaluate the impact of providing authors with an article template to be used in Microsoft Word and including links to examples of good reporting. Our hypothesis was that providing a template early in the writing process but under less controlled conditions than COBWEB might lead to more complete reporting compared to providing a reporting guideline recommendation alone.

## Methods

Before starting recruitment to the trial, we registered the study protocol in the Open Science Framework on 12 April 2020 [11] The trial is reported in accordance with the Consolidated Standards of Reporting Trials (CONSORT) statement [4].

### Design

We conducted a two-group parallel 1:1 randomized trial to evaluate whether sending authors an article template improved completeness of reporting of their manuscript before submission to a journal compared to a control intervention.

### Stakeholder involvement (PPI)

This section is reported in accordance with the GRIPP2 [12] reporting guideline for reporting stakeholder involvement in research.

To help us design the article template intervention, we recruited author stakeholders (*n* = 4) to participate in small focus groups where we aimed to identify which aspects of writing up research were most challenging for them. We used this information to finalise the features and format of the template which might overcome their challenges most effectively. Participants were recruited through EQUATOR Network social media channels, via a mailing list for people expressing interest in the project, and by contacting project partners. We targeted early-career researchers both from groups within University of Oxford Medical Sciences Division, and from countries with less well-resourced research institutions, such as Brazil.

We conducted the focus groups online due to the Covid-19 pandemic. This enabled a widening of our search for participants internationally. A table of responses collected during the focus groups can be accessed in the supplementary material [13]. Despite the wider scope for finding focus group participants, the numbers recruited were much smaller than the 30 participants we hoped for, so views may not have been representative of our target population. Two participants were from the UK, one a doctoral research fellow with a background in infectious microbiology, and one a research fellow writing up a Covid-related cohort study. One was a non-practising medical doctor from India planning a cohort study, and one was a practising clinician from Brazil interested in clinical study design, reporting quality and teaching. The stakeholder involvement influenced the wording and format of both the template and the control documents. All participants preferred the layout with the reporting item text and links to further information and examples integrated into the body of the template rather than as footnotes. The stakeholders had no influence over the choice of primary outcome measure.

The final article template was a Word document with headings and items from the appropriate reporting guideline for the study type specified by the author participant, integrated into a standard scientific research article framework (IMRaD). It also included links to explanations and examples of good reporting. The control was a Word document with a recommendation to use the appropriate reporting guideline, as above, and general advice on reporting.

### Participants

Research authors were eligible to participate if they were:

- over the age of 18 years,
- leading the writing of an original article reporting the following types of healthcare-related research study: randomized trial of a healthcare intervention (including behavioural or psychological treatment), observational cohort, case-control, or cross-sectional study (including nutrition or diet studies), systematic review of health intervention trials.
- intended to complete and submit the article to a medical journal or pre-print server in the next 6–12 months.

Research authors were not eligible to participate if they were:

- reporting findings exclusively from qualitative research.

### Recruitment

Participants were recruited via email, social media, and other digital channels, such as pop-up adverts and journal blogs, where we posted invitations to participate in the trial. We created a trial webpage (www.goodreports.org/grreat/) which included a description of the trial, and information on eligibility. Targeted potential participant communities included EQUATOR newsletter subscribers, Penelope.ai [14] users, GoodReports [7] users, the AuthorAID [15] community, researchers with registered studies on the ISRCTN database [16], students and research staff of universities and medical schools in over 40 countries and spanning five continents, and via journal blogs including BMJ Open [17] and BMC [18]. During the regular UK EQUATOR Centre training sessions and scientific meetings, we also mentioned the trial and invited authors to participate.

The incentive for agreeing to participate in the trial was the receipt of a free manuscript check and feedback report undertaken by reporting experts from the UK EQUATOR Centre (JdB and PL). Participants were informed that the assessors would check the completeness of the title, methods, and results sections of their final manuscript against the “gold-standard” reporting items appropriate for their study type. There was no other incentive offered.

### Consent and enrolment

Potential participants could access the participant information and online consent form via Oxford’s JISC online Survey tool. As part of the consent process, each potential participant was also asked to choose from a list of study designs and write a paragraph describing their study to help us confirm their eligibility. If there was any doubt, for example if the description didn’t match the study design they had selected, or was too brief, the lead investigator (CS) wrote to the potential participant to seek further information before confirming eligibility.

After participants had given consent, and their eligibility had been confirmed, CS asked them to complete an online form to collect baseline data including information about previous experience of research and of writing research articles for publication.

### Intervention: goodreports article template

Article templates were constructed by integrating items from seven reporting guidelines into a Word document with headings and reporting items which formed the framework for an academic article. The seven reporting guidelines were STROBE for cohort studies [5], STROBE for cross-sectional studies [5], STROBE for case-control studies [5], STROBE-nut [19], CONSORT [4], CONSORT-SPI [20], and PRISMA [21].

The article template included the main reporting guideline recommendation and a link to the checklist for that reporting guideline on the GoodReports website (e.g. checklist for STROBE cohort [22]). The template also included suggested headings and integrated the description text from each reporting guideline item. Hyperlinks from each reporting guideline item description to the GoodReports website where there were further explanations and examples of good reporting were made available. We also included links to other reporting guidelines and extensions related to the main recommended guideline, and general information on good reporting on the EQUATOR website.

The template instruction page also included a link to the SAMPL [23] guideline for basic statistical reporting of methods and results, and a link to the GRIPP2^12^ reporting guideline for reporting patient and public involvement. We did not assess adherence to either of these reporting guidelines.

### Control: reporting guideline recommendation

The control group received a document which included a recommendation to use the reporting guideline applicable to their study, and link to the database entry for that reporting guideline on the EQUATOR website. The control group were also provided with a link to the SAMPL reporting guideline, a link to the GRIPP2 reporting guideline, links to other related reporting guidelines, and general information on good reporting. The control was designed to mimic the type of advice on reporting usually contained in journal “instructions to authors” pages, although it gives more personalised help than would be typical.

The intervention and control documents were sent to participants as an email attachment. We did not require participants to acknowledge receipt. All participants were sent an email approximately every six to eight weeks for up to 12 months to check the status of their article, to encourage them, and to remind them to submit a copy of their manuscript for a completeness assessment before submitting it to a journal.

An example of the template intervention and the control document for a participant writing up a cohort study can be found in the supplementary material [13].

### Primary outcome assessment

For both randomized groups, the recommended reporting guideline was used by the outcome assessors to assess the primary outcome, completeness of reporting. Reporting completeness was assessed by evaluation of discrete reporting items from the title, methods, and results sections. Assessors evaluated the level of completeness of each item on the reporting checklist as (1) fully reported, (2) partially reported, (3) not reported/missing or (4) not applicable. An item scored 1 if it was fully and clearly reported, an item that were partially or unclearly reported received a score of 0.5. Items not reported or missing received a score of 0. Items which were not applicable were coded NA. To make assessments of completeness easier, many items were further split into subitems and counted individually for the purposes of the completeness scoring.

### Primary outcome data collection

A data collection form was created in REDCap for each of the seven reporting guidelines available to the author participants. Each form contained completeness scoring options for the reporting items and subitems from the title, methods, and results section of the article.

For each manuscript, the primary outcome was assessed and recorded in duplicate by co-investigators JdB and PL independently of each other and input directly into the Research Electronic Data Capture (REDCap) application (https://redcap.medsci.ox.ac.uk/). Additional data on use of reporting guidelines was recorded for each manuscript, such as whether the author had written about using a reporting guideline to prepare their manuscript, or whether participants included a checklist when sending us their manuscript. The assessors met to reach consensus on the primary outcome and to agree on the wording of feedback for each manuscript. The consensus and feedback record was used by CS to prepare a completeness feedback report which was sent to the author participant. CS then added randomization status and baseline data to the consensus record to create a complete baseline and results record for each participant. The feedback text was not included in the results data.

An example of an outcome assessment form, and corresponding feedback report for a case-control study can be found in the supplementary material [13]. The feedback text has been redacted.

### Baseline and follow-up data collection

These data were collected using Oxford’s JISC online Survey tool. Follow-up data about participants’ experience of writing their articles using the intervention or control, and journey through the publication process after participation in the trial was collected using an anonymous online form. Both forms “Supplementary materials_Baseline data collection form” and “Supplementary materials_Long term follow-up data collection form” are available in the supplementary information [13]. We asked permission to collect follow-up data during the consent process and again when we sent the feedback report. If participants confirmed they were happy to be contacted, we sent a link to the follow-up survey six months after we had sent the feedback report. The follow-up survey included a range of questions about their experience of using the GoodReports template or the reporting guideline recommendation, including whether they used any additional reporting guidelines beyond the one recommended, the usefulness of the completeness assessment report, and whether they submitted a checklist to the journal with their manuscript.

### Sample size calculation

The target sample size was 206 manuscripts (103 in each arm).

In a previous study, we compared reporting guideline adherence in 20 manuscripts before and after the lead author completed a reporting checklist as part of the journal submission process [1]. Five (25%) manuscript authors added information to reporting items in the methods section of their manuscripts after having completed the checklist. These five manuscripts went from 63% of items reported to 74% before and after receiving the GoodReports checklist, which is an absolute improvement of 11% points or a relative increase of 18% (11/63). We assumed that our control group authors, who were recommended a reporting guideline at an earlier stage of the writing process, would achieve a greater improvement in reporting than the pilot study authors.

For this trial, we compared completeness of reporting between two groups, rather than measuring whether information was added after exposure to a reporting checklist. Given the lack of previous research comparing two interventions designed to improve completeness of reporting, it was difficult to make a hypothesis about the expected effect to inform our sample size, Instead, we assumed that 20% of the control group would write a “perfect” report of their study, defined as reporting every item in the title, methods, and results sections of their manuscript completely. We aimed for a 25% target difference, meaning that we predicted that 45% of the intervention group authors would report every item completely. To achieve this difference with 90% power and a dropout rate of 30%, the sample size needed to be 206.

### Randomization

After confirming eligibility, participants were randomized to either the intervention or control arm using a computer-generated list of random numbers. The random sequence was created by GSC using R (version 4.1.0) statistical software using block randomization with random block sizes of 2, 4, 6, and 8.

On enrolment by completion of the baseline survey, the lead investigator (CS) assigned the participant a unique ID starting from GR001, GR002, GR003 etc. CS emailed the PI (GSC) with the message “Please randomize GR###” using the random sequence and GSC sent back a single word email with the assignment – ‘intervention’ or ‘control’. CS then emailed the intervention or control Word document to the participant according to the assignment. The participants were informed of their assigned group in the email message accompanying the Word document. The outcome assessors (PL and JdB) were blinded to the assignment.

### Analysis

For the primary outcome, we calculated a completeness ratio for each manuscript by dividing the number of completely reported items (receiving a score of 1) by the total number of relevant items (total of items and sub-items minus the items marked as not applicable). We decided retrospectively to analyse the 0.5 scores for a partially reported item in the analysis in two ways. In the original analysis, we coded a partially reported item as 0 in the statistical analysis, indicating it had not been reported. In the sensitivity analysis we coded a partially reported item as 1 indicating it had been reported. An item which was not applicable was labelled NA. NA items were not included when calculating the total number of reporting items in the title, methods and results section of the reporting guideline being used for the assessment. The completeness score for both analyses was the ratio of reported items to the number of relevant items. Statistical analyses were conducted with all consenting participants retained in the randomized arm forming an intention-to-treat population. For participants who did not return a manuscript, we were unable to assess reporting completeness and so they were excluded from the analysis. Participants who returned a manuscript formed our modified intention-to-treat population. The primary outcome was analysed using a two-sample t-test to compare the difference in mean completeness between the intervention and control group. Results of the t-tests were reported as mean differences with 95% confidence intervals and associated p-values. Analyses were carried out in R version 4.2.0.

### Exploratory analysis

Subgroup analyses were planned and specified in the protocol but due to the small sample size recruited these were not carried out.

### Changes from protocol

We deviated from the protocol in a few ways which did not materially affect the conduct of the trial. The main changes were:

- The randomization process was done manually rather than as an automated feature of the trial webpage.
- A menu of seven article templates and corresponding control documents was created by one of the research team (CS) rather than automatically generated.
- The templates and control documents did not attempt to combine items from more than one reporting guideline as this made the templates too long and unwieldy. Also, many of the important extensions we intended to use, such as TiDIER [24] and CONSORT Harms [25], had restrictive copyright and usage licences.
- The partially reported items (receiving a score of 0.5) were coded and contributed to the completeness scores compared in the analysis in two ways. In the original analysis partially-reported items were coded 0: not reported. In a second sensitivity analysis they were coded as 1: reported.
- We planned to have a random selection of manuscripts evaluated by a researcher independent of our team. However, as the number of manuscripts received was so low, we decided it would not be cost effective.

## Results

### Recruitment

**Fig. 1:**
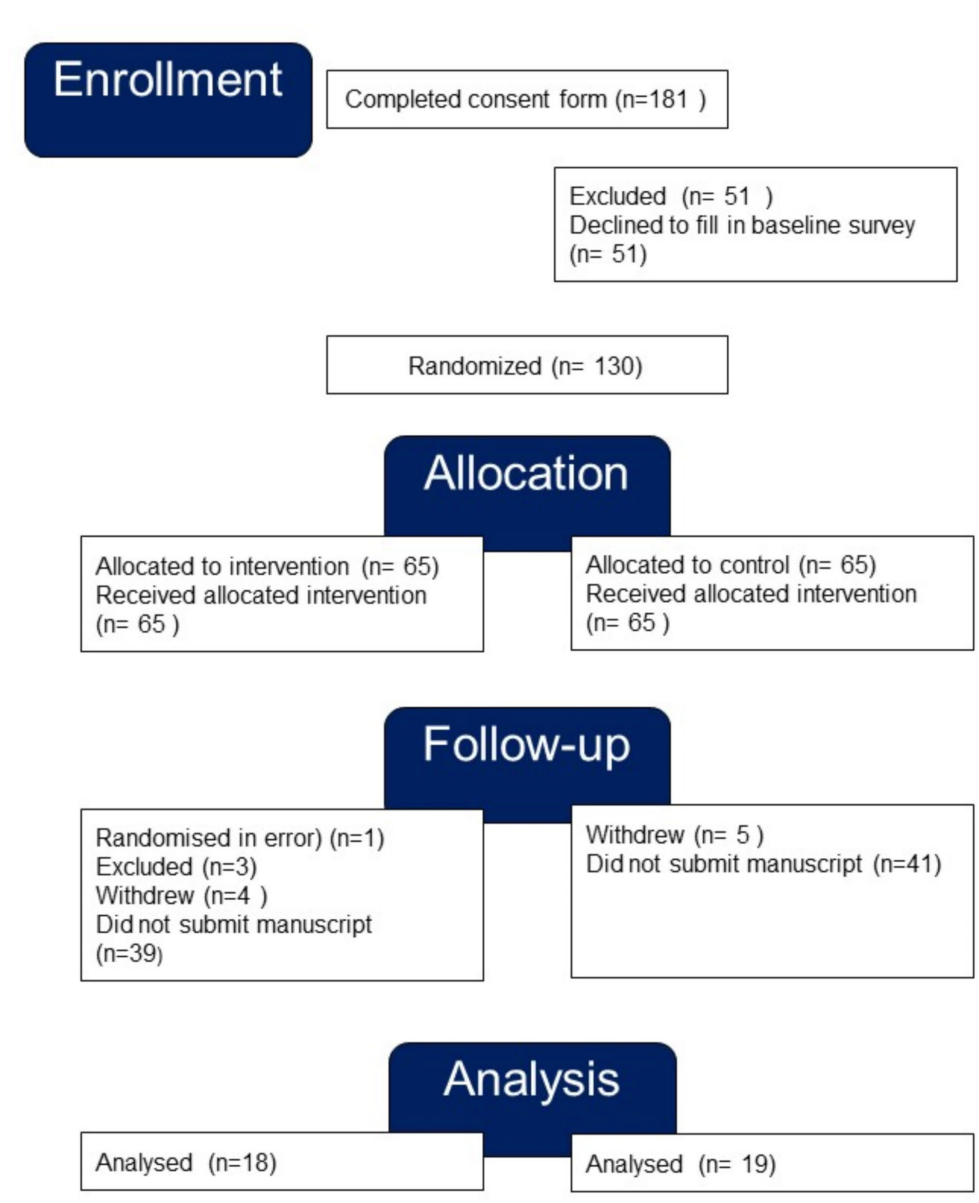
CONSORT flowchart

Enrolment and recruitment took place between 8 June 2021 and 31 December 2022. 181 people completed the consent form, and 130 (72%) completed the baseline survey and were randomized (Fig. 1). Recruitment reached 63% of the target sample size of 206. 65 participants were allocated to the intervention and 65 to the control group. See Table 1 for baseline characteristics.

**Table 1:** Baseline characteristics

Randomized	Submitted for assessment (primary analysis population)						Lost to follow-up					
Total	Total		Intervention		Control		Total		Intervention		Control	
n	n	%	n	%	n	%	n	%	n	%	n	%
130	37	28.5%					93	71.5%				
			18	48.6%	19	51.4%			47	50.5%	46	49.5%
Study type												
Case control	1	2.7%	0	0.0%	1	2.7%	5	5.4%	3	3.2%	2	2.2%
Cohort	4	10.8%	2	5.4%	2	5.4%	16	17.2%	9	9.7%	7	7.5%
Cross-sectional	14	37.8%	6	16.2%	8	21.6%	45	48.4%	28	30.1%	17	18.3%
Randomized trial	5	13.5%	4	10.8%	1	2.7%	8	8.6%	3	3.2%	5	5.4%
Systematic review	13	35.1%	6	16.2%	7	18.9%	18	19.4%	3	3.2%	15	16.1%
Nutrition/diet study	0	0.0%	0	0.0%	0	0.0%	1	1.1%	1	1.1%	0	0.0%
Trial of social/psychological intervention	0	0.0%	0	0.0%	0	0.0%	0	0.0%	0	0.0%	0	0.0%
Primary role												
Student	16	43.2%	7	18.9%	9	24.3%	38	40.9%	18	19.4%	20	21.5%
Not student/treating patients	15	40.5%	7	18.9%	8	21.6%	38	40.9%	21	22.6%	17	18.3%
Not student/not treating patients	6	16.2%	4	10.8%	2	5.4%	12	12.9%	7	7.5%	5	5.4%
Student question blank	0	0.0%	0	0.0%	0	0.0%	1	1.1%	0	0.0%	1	1.1%
Student and treating patients question blank	0	0.0%	0	0.0%	0	0.0%	4	4.3%	1	1.1%	3	3.2%
Research experience												
Less than 2 years	11	29.7%	4	10.8%	7	18.9%	28	30.1%	11	11.8%	17	18.3%
2–5 years	10	27.0%	6	16.2%	4	10.8%	37	39.8%	22	23.7%	15	16.1%
6–10 years	10	27.0%	6	16.2%	4	10.8%	12	12.9%	7	7.5%	5	5.4%
More than 10 years	6	16.2%	2	5.4%	4	10.8%	12	12.9%	6	6.5%	6	6.5%
Blank	0	0.0%	0	0.0%	0	0.0%	4	4.3%	1	1.1%	3	3.2%
NUMBER OF PREVIOUSLY PUBLISHED ARTICLES												
None	10	27.0%	6	16.2%	4	10.8%	21	22.6%	10	10.8%	11	11.8%
1–5 papers	14	37.8%	7	18.9%	7	18.9%	31	33.3%	15	16.1%	16	17.2%
6–10 papers	3	8.1%	3	8.1%	0	0.0%	10	10.8%	6	6.5%	4	4.3%
More than 10 papers	10	27.0%	2	5.4%	8	21.6%	26	28.0%	14	15.1%	12	12.9%
Blank	1		0		1		5	5.4%	2	2.2%	3	3.2%
NUMBER OF TIMES AS FIRST AUTHOR												
First time	10	27.0%	6	16.2%	4	10.8%	21	22.6%	11	11.8%	10	10.8%
1–5 articles	15	40.5%	7	18.9%	8	21.6%	47	50.5%	23	24.7%	24	25.8%
6–10 articles	5	13.5%	3	8.1%	2	5.4%	9	9.7%	4	4.3%	5	5.4%
More than 10	7	18.9%	2	5.4%	5	13.5%	12	12.9%	8	8.6%	4	4.3%
Blank	0	0.0%	0	0.0%	0	0.0%	4	4.3%	1	1.1%	3	3.2%
GEOGRAPHIC REGION												
Africa	7	18.9%	4	10.8%	3	8.1%	29	31.2%	18	19.4%	11	11.8%
Middle East/Asia/Oceania	11	29.7%	3	8.1%	8	21.6%	18	19.4%	10	10.8%	8	8.6%
Central/South America or Caribbean	3	8.1%	2	5.4%	1	2.7%	8	8.6%	1	1.1%	7	7.5%
Europe	12	32.4%	6	16.2%	6	16.2%	24	25.8%	14	15.1%	10	10.8%
North America	2	5.4%	2	5.4%	0	0.0%	3	3.2%	1	1.1%	2	2.2%
Australia/New Zealand	2	5.4%	1	2.7%	1	2.7%	3	3.2%	1	1.1%	2	2.2%
Blank	0		0		0		8	8.6%	2	2.2%	6	6.5%
LOCATION OF RESEARCH												
Local (regional)	14	37.8%	6	16.2%	8	21.6%	22	23.7%	13	14.0%	9	9.7%
Local (national)	13	35.1%	9	24.3%	4	10.8%	43	46.2%	23	24.7%	20	21.5%
Other nation (one site or multi-site)	0	0.0%	0	0.0%	0	0.0%	6	6.5%	4	4.3%	2	2.2%
International (multi-site, multi-country)	0	0.0%	0	0.0%	0	0.0%	6	6.5%	3	3.2%	3	3.2%
N/A (e.g. systematic review)	10	27.0%	3	8.1%	7	18.9%	11	11.8%	2	2.2%	9	9.7%
Blank	1	2.7%	0	0.0%	1	2.7%	5	5.4%	2	2.2%	3	3.2%
LANGUAGE												
English first language	10	27.0%	5	13.5%	5	13.5%	28	30.1%	16	17.2%	12	12.9%
English not first language	27	73.0%	13	35.1%	14	37.8%	61	65.6%	30	32.3%	31	33.3%
Blank	0	0.0%	0	0.0%	0	0.0%	4	4.3%	1	1.1%	3	3.2%
SEX												
Female	15	40.5%	10	27.0%	5	13.5%	30	32.3%	14	29.8%	16	34.8%
Male	22	59.5%	8	21.6%	14	37.8%	59	63.4%	32	68.1%	27	58.7%
Blank	0	0.0%	0	0.0%	0	0.0%	4	4.3%	1	1.1%	3	3.2%

41 participants (32%) delivered completed manuscripts for assessment between September 2021 and June 2023. Four were excluded from the analysis. One was excluded because they had been randomized in error; CS failed to notice before randomization that they had replied “no” to several of the questions on the consent form. A further three were excluded, one because they sent a report of a feasibility study for a randomized trial instead of the cohort study they described on enrolment, one because they submitted a case-cohort study instead of a cohort study they declared they would do, and one submitted a manuscript which didn’t match either the study design they had specified (cross-sectional study) or the topic of the summary they submitted with the consent form. The average time to deliver the manuscript was six months for both the intervention and the control group. The median time to deliver was five months ranging between two and 17 months, and six months ranging between one and 10 months respectively.

### Analysis of the primary outcome

Thirty-seven manuscripts (18 intervention, 19 control) were assessed for completeness and included in the intention-to-treat analysis of the primary outcome of reporting completeness. This represented a drop-out rate of 72% (93/130). The assumed drop-out rate used to calculate the sample size was 30%.

Analysis including partially reported items as not reported (original analysis).

The mean (standard deviation) proportion of completely and partially reported items from the title, methods, and results sections of the 37 assessed manuscripts (primary outcome) was 0.57 (0.18) in the intervention group and 0.50 (0.17) in the control group. The mean difference between the two groups was 0.069 (95% CI -0.046 to 0.184; *p* = 0.231).

Sensitivity analysis including partially reported items as reported.

The mean (standard deviation) proportion of completely and partially reported items from the title, methods, and results sections of the 37 assessed manuscripts (primary outcome) was 0.75 (0.15) in the intervention group and 0.71 (0.11) in the control group. The mean difference between the two groups was 0.036 (95% CI -0.127 to 0.055; *p* = 0.423).

There was no significant difference in reporting completeness between groups in either the original or the sensitivity analysis.

The additional observational data collected during the completeness assessment, such as whether the manuscript reported the use of reporting guidelines, or whether a reporting checklist was submitted with the manuscript can be seen in Table 2.

**Table 2:** Use of reporting guidelines by participants

Total	Intervention		Control	
n	n	%	n	%
37	18	48.6%	19	51.4%
Reported using recommended reporting guideline	10	27.0%	8	21.6%
Submitted GoodReports version of checklist	3	8.1%	0	0.0%
Submitted version of checklist from original paper	0	0.0%	3	8.1%
Reported using GRIPP2 (public involvement)	0	0.0%	0	0.0%
Reported using SAMPL (statistics)	1	2.7%	0	0.0%
Reported using other reporting guidelines	2	5.4%	1	2.7%

### Follow up survey

Between April 2022 and October 2023, the anonymous follow-up survey was sent to the 37 participants who had had their manuscript assessed and been sent a feedback report. Nineteen participants completed the follow up survey, 61% (11/18) in the intervention group and 42% (8/19) in the control group. Their self-reported use of the recommended reporting guideline was 56% (10/18) in the intervention group compared with 42% (8/19) in the control group. All respondents who received the feedback report before submitting their manuscript to a journal (18/19) reported they had changed their manuscript either a little or a lot.

As only 19 people submitted the follow-up survey, we didn’t statistically analyse the responses. The participants’ responses from a menu of choices on their experience of writing their manuscript, and their progress through the publication process is reported in Table 3.

**Table 3:** Participants’ reported experience of writing and submitting their manuscript for publication

TOTAL SUBMITTED MANUSCRIPTS		Intervention	Control
*n* = 37		*n* = 18	*n* = 19
Follow up survey responses			
*n* = 19		*n* = 11	*n* = 8
If you used template what was most useful	The reminder to use reporting guidelines	1	1
	The main reporting guideline recommendation	3	5
	The links from the reporting guideline items to explanations and examples of good reporting	2	n/a
	The headings and items from the recommended reporting guideline	4	n/a
	The links to other reporting guidelines and resources	n/a	2
Reported use of reporting guidelines	I used the recommended reporting guideline only	8	6
	I used the recommended reporting guideline and one or more additional guidelines	1	1
	I didn’t use reporting guidelines	1	0
	Not sure/can’t remember	1	1
Usefulness of feedback report	Very useful	6	5
	Moderately useful	5	3
Did you change your article?	I changed it a little	7	5
	I changed it a lot	4	2
	I submitted my article before I received the feedback report	0	1
Submitted to a journal	Yes	8	8
	No, plan to submit in future	3	0
	No, no plans to submit in future	0	0
Submission of checklist with manuscript	Yes	7	3
	No	1	5
How many journals submitted to	One	6	4
	Two	1	1
	Three	0	2
	More than three	1	1
Initial Decision	Rejection	3	1
	Send for peer review	4	7
	No response yet	1	0
Outcome of peer review	Rejection	0	1
	Request for minor amendment	2	4
	Request for major amendment / revise and resubmit	3	2
	Can’t remember/unsure	0	1
Final decision	Accept for publication	1	4
	Decision not yet made	5	3
	Reject	2	0

Lightly edited free text responses on participants’ responses are available in the supplementary material.

## Discussion

GRReaT sought to test whether providing customized reporting guidance within writing templates for use throughout the writing process resulted in clearer and more complete reporting than only giving advice on which reporting guideline to use.

Despite a range of targeted attempts to boost recruitment repeated throughout the study period, the trial failed to reach the pre-determined sample size, and there was a larger than anticipated drop-out rate. The small difference observed between the groups was not statistically significant, and we were unable demonstrate that providing authors with customized article templates including items from reporting guidelines improved the completeness of reporting. The challenges we faced in carrying out a study of an intervention introduced during the article writing process have provided several learning points for others wishing to investigate similar interventions, which we discuss below.

### Strengths

#### Real life context

All previous randomized trials of interventions to improve reporting have been conducted as part of a journal editorial and peer review system [27, 28, 29, 2, 30, 31, 32, 33], or outside a real-world context [10]. This trial recruited participants directly without an intermediary recruitment mechanism such as a journal submission system, or endorsement by a research institution or regulator. If the required sample size had been reached and the drop-out rate much lower, the results would have been generalisable to researchers at the beginning of their writing journey where reporting guidelines are potentially a more useful tool.

#### Participants offered feedback

Studies have suggested that passive interventions such as a request that authors or peer reviewers use reporting guidelines or checklists, are ineffective ^2 30 33^. Possible reasons for this are discussed in a recent thematic analysis [8]. These could include authors lacking time or motivation, not fully understanding the guidance, or finding the checklist but not the explanation or examples. Interventions showing most promise have been active, providing additional review and feedback to authors [27, 28, 29, 34 31]. This is the first randomized trial of a reporting tool - in this case an article template - designed to be used from the beginning of the writing process. It explicitly fills one of the research gaps identified in a recent scoping review [9] which calls for the development and testing of an intervention which includes checks of reporting guidance adherence and provides feedback to authors outside a journal editorial system. All participants knew to expect a review of adherence to reporting guidelines, so we presumed both the intervention and control groups would have the same incentive to follow the guidance made available to them. However, the intervention itself is still passive as we didn’t attempt to collect data on the effect of the feedback report. Outside the trial, there would be no incentive to use the template writing aid, unless introduced as a standard part of regulatory, editorial and publication systems.

#### Feasibility

Although our study was not able to answer the research question about whether the intervention led to a meaningful improvement in reporting quality, we demonstrated it was feasible to test it in a randomized trial. We originally intended to develop the GoodReports website software to generate and deliver the template intervention (or control) automatically. This turned out to be technically complex and not achievable in the time available. However, manual generation and delivery of the intervention, and the method of checking adherence of submitted manuscripts using reporting experts have been proved to be feasible.

#### Scalability

The GoodReports website [7] currently contains a library of online downloadable checklists, explanations of reporting items, and examples of good reporting. There is therefore an existing framework in place which could be developed further to include more reporting guidelines. It would also be a relatively simple extension of the website functionality to enable “self-service” and allow authors to generate a range of different formats for reporting guidance, such as article templates, automatically.

#### Direct engagement with authors

Recruiting participants directly rather than via a journal editorial system, whilst challenging and labour intensive, allowed a unique insight into the individual author experience of the intervention which has not been explored in previous trials. The incentive of a free check by reporting experts from the UK EQUATOR Centre irrespective of group allocation was designed to attract those who were less confident and experienced; the group of researchers who most need to be aware of reporting guidelines and be able to access help with using them effectively.

#### Responsive to future research

Whilst writing up the results of this study, we discovered a protocol for a randomized trial to test article templates based on PRISMA and extensions for reporting systematic reviews [26]. We changed our method for calculating the completeness score to make the results of this trial more easily comparable with the results of this future trial.

### Limitations

The key limitation of the trial was the failure to recruit the required sample size and the high drop-out rate.

#### Author stakeholder involvement

We were only able to involve four authors in focus groups to inform the design of the template. This was far short of the 30 we had aimed for so views may not have been representative of our target population.

#### Recruitment

The trial was running throughout the Covid-19 pandemic so health researchers would have had many other priorities during that time which could have contributed to the low recruitment and high level of attrition. We had originally intended to partner with two journals on recruitment, but realised we would have no chance of recruiting the required numbers if we didn’t widen the pool of potential participants considerably. Our more open recruitment strategy meant we had, in principle, the potential to recruit a larger and more representative sample of participants. However, it was more difficult than expected to recruit people. This could have been because we were unable offer the reassurance that participation was taking place in the context of a journal’s editorial system. If we had recruited via journal submission systems, it may have created the impression that participants’ manuscripts were more likely to be accepted, and improved recruitment rates. However, unless this impression was the truth, it could be interpreted as coercion and would be unethical.

Although we made no record of where participants were recruited from, we observed that the most successful strategy, tried towards the end of the recruitment period, seemed to be identifying and approaching individual research leaders at medical schools and health research institutes around the world, targeting Commonwealth countries. We then emailed each leader personally, emphasising the fact it was a University of Oxford research study, and asked them to publicise the opportunity to the students and junior researchers under their supervision. The downside of this approach was the time taken to identify leaders and contact people individually. The supplementary material includes the table of university research leaders and their contact details identified from publicly available sources during this process [13].

Some potential participants, even with the offer of a free manuscript check by University of Oxford reporting experts at the UK EQUATOR Centre, may have been unwilling to take the unusual step of committing to sharing a manuscript draft outside a journal editorial process. This reluctance to share a manuscript was mentioned explicitly by a colleague and enthusiastic proponent of the trial in Japan who had tried to persuade their students to participate. One way we may have overcome scepticism would have been to recruit local EQUATOR advocates to translate and edit the participant information to provide reassurance to potential participants tailored to their most common concerns.

#### Drop-out rate

Despite the incentive of a free reporting check from experts at the UK EQUATOR Centre, the dropout rate was more than double what we expected.

Participants may have dropped out because we hadn’t communicated well enough how to use the template, or, in the control group, didn’t understand the purpose of reporting guidelines or how to use the reporting guideline publications. They may have been too far along with their writing and either didn’t have time to figure out how to apply the guidance to their work retrospectively or felt it would make their writing too verbose. They may not have recognised or believed in the benefits of using reporting guidance we attempted to communicate to them. In an extreme case, one participant dropped out because they had assumed that the study team would liaise with the editorial staff of a journal on their behalf including payment of the publication fee.

Researchers conducting similar trials in the future could conduct surveys or interviews to explore the reasons for lack of engagement, and proactively attempt to mitigate these and other potential factors to keep participants incentivised and engaged.

#### Masking of outcome assessors

Although the assessors were blinded to the group allocation, there would have been several clues to reveal the allocation. For example, participants in the control group were advised to use the published version of the reporting guideline and submit the corresponding checklist with their manuscript. Those in the intervention group were directed to the online version of the reporting guideline and the checklist on the GoodReports website. Because the GoodReports checklists looked different to the original, outcome assessors could have deduced participant allocation. This was not an issue, as few of the participants submitted a reporting checklist with their manuscripts. If time and resources had allowed, we could have recruited additional assessors unaware of the design of the intervention and control and this would have ensured masking was better maintained.

#### Difficulty reaching consensus

Despite efforts to minimise the potential for disagreement on completeness assessments, discussions to reach consensus were often lengthy, and consensus meetings took at least one hour in average per manuscript, with assessors discussing only the items where they disagreed. Sometimes the disagreement points were resolved quickly by one assessor showing the other where the information was in the manuscript. But frequently the discussions were around whether a piece of information was clear and completely reported according to the reporting guideline — not according to assessors’ preference or taste. This required assessors to consult reporting guidelines’ explanation and elaboration documents frequently, which may indicate a level of ambiguity in the way reporting guidelines are written and interpreted, both by both authors and reporting experts.

#### No measurement of outcomes after feedback

Although we conducted an anonymous follow-up survey, the numbers completing it were low and susceptible to recall bias. However, all respondents from both the intervention and the control group reported they had changed their manuscript in response to the feedback report. The only way to confirm would be to identify the published articles and compare them with the manuscripts submitted to us as part of the trial. We had originally intended to do this, but the number of participants was too small to make it worthwhile to aggregate the data and preserve anonymity. This would be a useful outcome measure in future trials with more participants and could be set up easily by setting alerts for article titles in Google Scholar.

### Considerations for future research

Although this study didn’t show templates to work, we think they should be considered for further adaptation, development and testing to help bring the importance of clear and complete reporting higher up the agenda in research culture. Some of the changes needed to scale up the availability, testing and implementation of templates are discussed below.

#### Study design

In the early days of recruitment, we didn’t publicise the opportunity to participate in the trial too widely as we were anxious to avoid cross-contamination caused by people in the control group working with people in the intervention group. In future, this risk could be avoided by conducting a cluster-randomized trial. This would mean all authors in the same institution would receive either the template or the control which would avoid the worry of cross-contamination. Recruitment could then be conducted via health research leaders at institutions whose endorsement of the trial might encourage participation.

#### Remove restrictive usage licences

In this trial we could only offer article templates for reporting guidelines without restrictive usage licences. A database of reporting guidelines licences made public in 2022 [35] showed that approximately half of published reporting guidelines or extensions are published with restrictive licences (i.e., not CC-BY), which allow reading but not reusing materials. This meant we were unable to include items from these reporting guidelines in the article templates without permission from the publisher, which might also entail paying a yearly licence fee. This prevented us from being able to generate templates for some common study types, or to integrate items from important extensions, such as CONSORT Harms [25].

Another research team are now exploring a similar way to deliver this type of novel active intervention for systematic reviews using PRISMA and PRISMA extensions [26]. They have already encountered the issue of restrictive copyright licences which may hamper their efforts to harmonise and deliver PRISMA guidelines and extensions in this way. We hope publishers will be persuaded to waive usage fees and restrictions on derivative products. Otherwise, it will be prohibitively expensive to scale up this type of intervention to give comprehensive support for all study types [36].

#### Develop guidance for reporting all common study designs

A trial testing the use of statistical reviewers to improve reporting [28] observed that the pre-post study (also known as a before-after study) is a very common study design with no reporting guideline. It would be useful to devise an additional article template for this and other common study designs which don’t yet have their own reporting guidelines.

Future interventions designed to increase adherence to reporting guidelines and improve health research reporting should not limit themselves to one reporting guideline. Even established reporting guidelines, such as STROBE, CONSORT and PRISMA, do not apply to all common study types, particularly those conducted by less experienced or less well-resourced researchers, such as before-after studies [28].

#### Help with identifying the appropriate guidance

Our original plan was to improve the GoodReports reporting guideline decision tree tool to help researchers do this. However, when we realised there were only a limited range of templates we could offer because of restrictive usage licences, we decided to rely on the potential participants to self-exclude if their study design was not listed as an option. As observed in previous studies, researchers can occasionally not know the type of study they’ve carried out [2, 37] or they have done a study where it is difficult to identify an appropriate reporting guideline [1]. This was particularly evident with observational studies. There was also a tendency for authors to miscategorise their study design. This could have been because they were keen to take part in the trial and receive expert feedback on their manuscript.

#### Increase access to pre-submission checks and feedback

Despite the existence of reporting guidelines, and the fact most journals endorse their use, submitted manuscripts are still, in general, reported poorly. This makes the job of editors and peer reviewers considerably more challenging. A challenge would be to resource the checking of adherence and the provision of feedback outside a trial. This is cost which is unlikely to be borne by journals across the board. An intervention to improve reporting would be more likely to be adopted by the whole research community if the burden didn’t fall on individual journal editors and peer reviewers to check adherence and provide feedback on reporting.

More of the responsibility for full and transparent reporting could be borne by funders and regulators. Guidance and feedback could also be given in advance of manuscript submission by reporting experts within the authors’ institutions, or by EQUATOR Network educators and ambassadors. These innovations would reduce the burden on editors and peer reviewers, allowing them to concentrate on the quality of the research itself. There may also be a place for reporting guidance to be integrated into, or at least signposted in new open publishing platforms, such as Octopus [38].

## Conclusion

There are numerous barriers to the use of reporting guidelines, many of which contributed to the difficulty in recruiting and retaining enough participants to answer our trial question. More effort and resources need to be invested incentivising the use of reporting guidelines, as well as making them more accessible and user friendly.

Our work has highlighted that there is no single solution to improving reporting of health research studies. The research community needs to work together towards systemic change. The commitment to change should come from all stakeholders, at all stages of writing about research, from conception and planning, through to reporting and dissemination of the results. Those who develop reporting guidelines need to be as aware of the problems of implementing the guidelines they produce, and as committed to change as all other stakeholders.

## Data Availability

The data for the primary outcome, the code used to analyse it, and the supplementary materials are accessible from the Open Science Framework osf.io/5br9213.

## Declarations

### Registration

We are committed to open science practices. The GoodReports Randomized Trial (GRReaT) protocol is registered on the Open Science Framework (osf.io/d8u76).

## Abbreviations

EQUATOR: Enhancing Quality and Transparency of Health Research
PRISMA: Preferred Reporting Items for Systematic reviews and Meta-Analyses
CONSORT: Consolidated Standards of Reporting Trials
CONSORT-SPI: Reporting randomised trials of social and psychological interventions
STROBE: STrengthening the Reporting of OBservational studies in Epidemiology
STROBE-nut: Strengthening the Reporting of Observational Studies in Epidemiology—Nutritional Epidemiology
